# Red light mediates the exocytosis of vasodilatory vesicles from cultured endothelial cells: a cellular, and ex vivo murine model

**DOI:** 10.1007/s43630-023-00522-1

**Published:** 2024-01-26

**Authors:** Dorothee Weihrauch, Agnes Keszler, Grant Broeckel, Eva Aranda, Brian Lindemer, Nicole L. Lohr

**Affiliations:** 1 Division of Cardiovascular Medicine, Department of Medicine, Medical College of Wisconsin, Milwaukee, WI USA; 2 Department of Anesthesiology, Milwaukee, WI USA; 3 Department of Plastic Surgery, Milwaukee, WI USA; 4 Cardiovascular Center, Medical College of Wisconsin, Milwaukee, WI USA; 5 Clement J Zablocki VA Medical Center, Milwaukee, WI USA; 6 Present Address: Cardiovascular Institute, University of Birmingham, Alabama, USA

**Keywords:** Red-light therapy, Photobiomodulation, Vasodilation, Exocytosis, *S*-Nitrosothiol

## Abstract

We have previously established that 670 nm energy induces relaxation of blood vessels via an endothelium derived *S*-nitrosothiol (RSNO) suggested to be embedded in vesicles. Here, we confirm that red light facilitates the exocytosis of this vasodilator from cultured endothelial cells and increases ex vivo blood vessel diameter. Ex vivo pressurized and pre-constricted facial arteries from C57Bl6/J mice relaxed 14.7% of maximum diameter when immersed in the medium removed from red-light exposed Bovine Aortic Endothelial Cells. In parallel experiments, 0.49 nM RSNO equivalent species was measured in the medium over the irradiated cells vs dark control. Electron microscopy of light exposed endothelium revealed significant increases in the size of the Multi Vesicular Body (MVB), a regulator of exosome trafficking, while RSNO accumulated in the MVBs as detected with immunogold labeling electron microscopy (1.8-fold of control). Moreover, red light enhanced the presence of F-actin related stress fibers (necessary for exocytosis), and the endothelial specific marker VE-cadherin levels suggesting an endothelial origin of the extracellular vesicles. Flow cytometry coupled with DAF staining, an indirect sensor of nitric oxide (NO), indicated significant amounts of NO within the extracellular vesicles (1.4-fold increase relative to dark control). Therefore, we further define the mechanism on the 670 nm light mediated traffic of endothelial vasodilatory vesicles and plan to leverage this insight into the delivery of red-light therapies.

**Graphical abstract:**

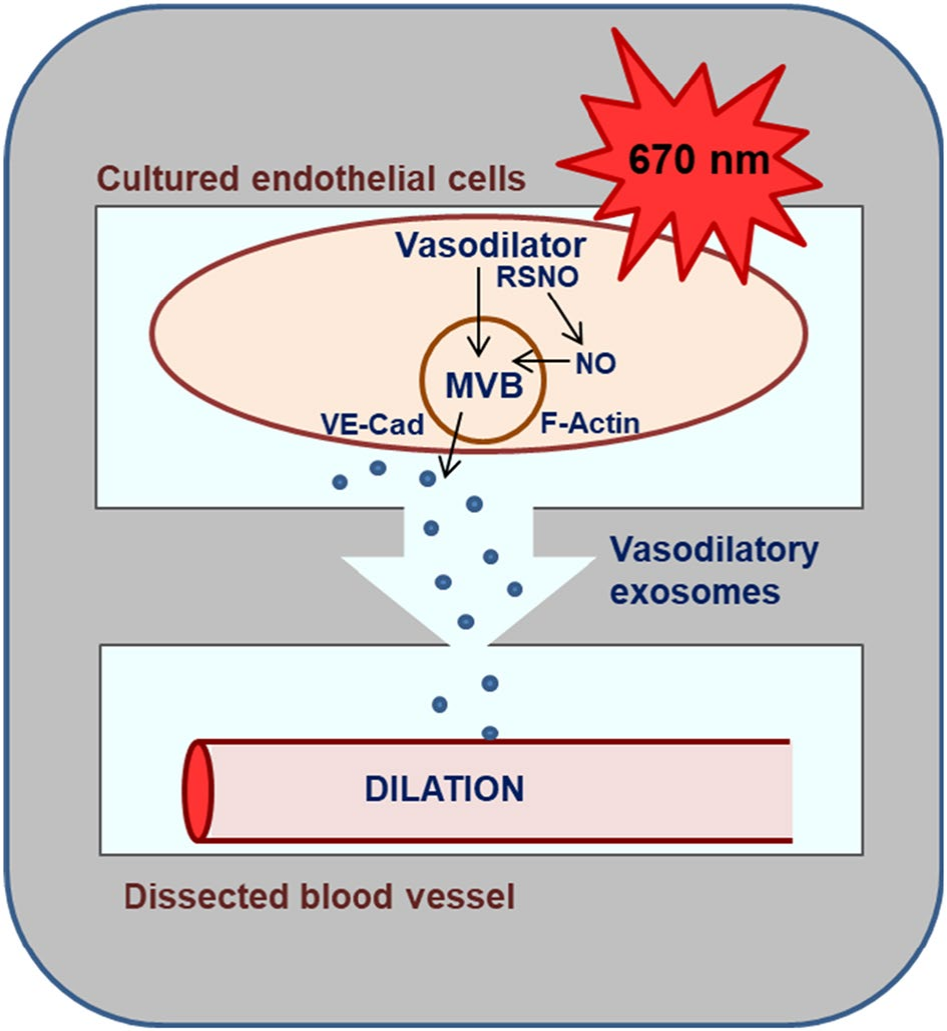

## Introduction

Photobiomodulation, or therapeutic application of laser or LED light in the red and near infrared region of the electromagnetic spectrum, is a promising modality to efficiently treat numerous pathological conditions [1–3]. In contrast to surgical solutions, red light treatments can be applied noninvasively in a home-based setting, and unlike drug therapies, it is considered side effect free. The mechanism through which light exerts its effect is complex and highly dependent on the targeted biological chromophores. Moreover, a major limitation to expanding its clinical use is the identification of multiple action spectra and the variable mechanisms by which the different light frequencies act [4–7], e.g., 830 nm energy was successful to reduce infarct size in dog and rat model through increasing mitochondrial respiration and ATP synthesis [4], while 670 nm was advised to reduce platelet aggregation via a nitric oxide (NO) and *S*-nitrosothiol (RSNO)-dependent mechanism [7].

Our previous investigations support the 670 nm wavelength to benefit vascular disorders, e.g. endothelial dysfunction when the synthesis of the critical relaxing factor NO is limited [8]. We suggested a mechanism by which 670 nm light improves the circulating blood flow by initiating exocytosis of endothelium derived vasodilatory substances, such as RSNO and presumably other NO-derived sources embedded in microvesicles. These vasodilatory substances are stable for at least 30 min, which suggests a partial sequestration of NO resulting in slower decomposition and increased stability [9, 10]. The novelty of these earlier findings necessitates a deeper understanding of the formation and movement of these vesicles and the role of 670 light in these processes. In our present work, we hypothesized that vesicles exiting the endothelial cells are active and able to enter dissected blood vessels to induce dilation, and the secretory materials transported through the cellular endomembrane system accumulate in the late endosomes or Multi Vesicular Bodies (MVB) before leaving the cell [11]. MVBs are the organelles where the versatile cargo is sorted and the biogenesis of exosomes occurs followed by their transport to the plasma membrane to exit and be ready for internalization by the recipient cells [12, 13]. The dynamics of membrane fusion during exocytosis are largely controlled by cytoskeletal filamentous actin (F-actin) which facilitates the secretion and subsequent uptake of vesicles by increasing the plasma membrane tension [14, 15]. The endothelial origin of the exiting vesicles can be recognized by specific markers e. g. VE-cadherin.

Using bovine aortic endothelial cells (BAECs) as a model, we investigated the effect of 670 nm light on formation, content, and exocytosis of vasodilatory vesicles, and how these vesicles trigger dilation of facial arteries dissected from C57Bl6/J mice.

## Materials and methods

### Materials

F-actin antibody was bought from Abcam (Waltham, MA). *S*-Nitrosocysteine (Cys-SNO) antibody was Creative Diagnostics (Shirley, NY) product. VE-cadherin antibody and Alexa 488 or 610 conjugated secondary antibodies were obtained from Santa Cruz Biotech (Dallas, TX). Lysobisphosphatidic acid (LBPA) antibody was ordered from EMD Millipore (Burlington, MA), and DAF-FM DA from Genaxxon bioscience GmbH (Ulm, Ge). DAPI stain were purchased from ThermoFisher Scientific (Waltham, MA). All other materials employed were Sigma-Aldrich (St. Louis, MO) products.

### Light source

The light sources (manufactured by Quantum Devices Inc, Barneveld, WI) used LED emitters at 670 nm wavelength. The power supply (same manufacturer) operated with adjustable output. Power output was determined with X97 Optometer (Gigahertz Optic Gbmh, Turkenfeld, Germany) before starting the irradiation. The cell culture dishes, or the chamber slides were placed directly on the surface of a brick type light platform containing multiple LED emitters. The applied dose was selected upon dose response-based optimization in previous experiments [16].

### Cell culture

BAECs (Strauss Veal, Franklin WI) were cultured under standard conditions using media containing RPMI (ThermoFisher; Waltham, MA), 20% FBS (ATCC; Manassas, VA), 2 mM L-glutamine, and 0.5% Pen/Strep. All plates and slides were treated with 2% gelatin prior to seeding. All experiments were performed using passages P5-P9. Cells were plated on 100 mm TPP cell culture dishes (Midwest Scientific, Valley Park, MO) and 4-well chamber cell culture slides (CELLTREAT Scientific Products, Pepperell, MA).

### Ozone-based chemiluminescence

RSNO was detected with triiodide-dependent ozone-based chemiluminescence [17], a sensitive and specific method to detect free NO, based on its oxidation with ozone to **˙**NO_2_ gas in excited electronic state, followed by a shift to ground state accompanied by photon emission (chemiluminescence (CL)). Acetic triiodide solution is used to liberate NO from its precursors such as nitrite and RSNO. Medium over the cells was replaced with physiological saline solution (PSS) used for pressure myography and irradiated (670 nm, 10 mW/cm^2^, 10 min) by placing the dish directly on the surface of the light platform. Then, the cells were lysed in 50 mM potassium phosphate/1 mM DTPA/50 mM NEM pH 7.4. Sulfanilamide ((100 mM in 2 N HCL) 10% (v/v)) was added to the samples to remove nitrite, while presence of RSNO was verified by measuring the samples after incubation with HgCl_2_ ((100 mM) 10% v/v)) which decomposes RSNO. The RSNO levels were calculated as a difference of CL signal before and after HgCl_2_ addition. Quantification was performed based on the detector response for known amounts of *S*-nitrosoglutathione.

### Pressure myography

Vasodilation was measured with pressure myography [18]. All experimental procedures and protocols used in this investigation were reviewed and approved by the Animal Care and Use Committee of the Medical College of Wisconsin. Furthermore, all conformed to the *Guiding Principles in the Care and Use of Animals* of the American Physiologic Society and were in accordance with the *Guide for the Care and Use of Laboratory Animals*. Segments of facial arteries (160–260 μm ID) from C57Bl6/J mice were transferred to a water-jacketed perfusion chamber and cannulated with two glass micropipettes at their in-situ length. The arteries were bathed in PSS maintained at pH 7.4 and 37 °C. The micropipettes were connected to a reservoir filled with PSS and the arteries were pressurized to 60 mmHg. The internal diameter of the arteries was measured with a video system composed of a stereomicroscope (Olympus CK 40), a charge-coupled device camera (Panasonic GP-MF 602), a video monitor (Panasonic WV-BM 1410), and a video measuring apparatus (Boeckeler VIA-100). After a 60-min equilibration period, the arteries were pre-constricted by ∼50% of their resting diameter with a thromboxane A_2_ analog, U-46619. The bath was replaced with the PSS collected over the irradiated cells. Vasodilator responses to papaverine (10^−4^ M) were determined and expressed as percent of maximal relaxation relative to U-46619 pre-constriction, with 100% representing the passive baseline diameter with papaverine.

### Flow cytometry

For NO measurements, 100 mm plates with 90–95% confluent BAECs were incubated with DAF-FM for 1 h at 37 °C. After washing, the media was replaced by fresh 2 ml DPBS, and the cells were irradiated (670 nm, 10 mW/cm^2^, 10 min), and the supernatant was collected. Supernatant from five plates were pooled and centrifuged first at 2000×*g* for 8 min to remove cellular debris and then the supernatant was further centrifuged at 100,000×*g* for 1 h at 4 °C. The isolated vesicles were resuspended in 500 µl of DPBS and incubated with 5 µl of FM 5–95 for 30 min at 4 °C in the dark.

For VE-Cadherin measurements, samples were incubated in 2.5 µl of VE-Cadherin antibody for 1 h at 4 °C and centrifuged at 100,000×*g* for 1 h at 4 °C. Supernatant was removed, and vesicles were re-suspended in 500 µl of fresh DPBS. Samples were then incubated with 5 µl of FM5-95 for 30 min at 4 °C in the dark. After FM5-95 incubation, all samples were centrifuged at 100,000×*g* for 1 h at 4 °C. Isolated vesicles were resuspended in 500 µl fresh DPBS.

Negative control vesicles consisted of a 50/50 mixture of light treated and dark control unstained vesicles, with a total volume of 500 µl as well. Samples were analyzed using a BD LSRFortessa X-20. AF488 and PE-Cy7 optimal intensities were then calibrated using the unstained control so that all events were within the visual field and had a negative peak fluorescence at ≤ 10^3^ on both the AF488 and PE-Cy7 lasers. 100–200 µl of each sample were acquired at approximately 200 events/s. Data analysis was performed using FlowJo software.

### Immunofluorescence

Typically, cells were cultured on coated 4-well chamber slides, washed, irradiated in HBSS at 670 nm, 50 mW/cm^2^ for 2 min, permeabilized in 0.5% TritonX (5 min), then incubated with recommended dilution of appropriate primary antibodies (30 min at 37 °C), then Alexa 488 or 610 conjugated secondary antibodies were applied (1:1000 dilution, 30 min at 37 °C). Nuclei were stained with DAPI (1:1000 dilution, 5 min). Slides were mounted with Shur/Mount medium (Triangle Biomedical Sciences, Durham, NC) and cover slipped. Images were captured and quantified with a fluorescent Nikon Eclipse Ti2 microscope using NIKON Element software.

### Electron microscopy

According to an established method [19], cells were fixed in 2.5% glutaraldehyde in 100 mM sodium cacodylate buffer at room temperature for 1 h. Washed in cacodylate buffer, scraped and pelleted, then cell pellets were post fixed in 1% osmium tetroxide on ice for 1 h. Cell pellets were processed through methanol series and acetonitrile into epoxy resin for overnight polymerization. Polymerized resin blocks were sectioned (RMC PTXL) 60 nm thick. Sections were stained with uranyl acetate and lead citrate and viewed in Hitachi H600 TEM. Images were captured on Hamamatsu digital camera running AMT imaging software.

For immunogold labeling, cells were processed according to published method [20]. Briefly, they were washed in 100 mM Sorensen’s phosphate buffer, scraped and pelleted. Pellets were aliquoted into 100 µm planchettes and high pressure frozen (Leica EMPact II). Vitrified cell samples were transferred to a freeze substitution apparatus (Leica EM AFS II) at − 90 °C into freeze substitution medium containing 2% (w/v) uranyl acetate, 10% (v/v) methanol, 2% (v/v) distilled water in acetone. After substitution warming process, samples were washed with dry acetone at − 50 °C and infiltrated with increasing concentrations of Lowicryl HM20 dissolved in acetone to pure HM20 over 10 h then polymerized by UV light at − 50 °C for 48 h. Polymerized samples were warmed slowly to 20 °C. Ultrathin (70 nm) sections were cut, mounted on formvar-coated copper grids and immune-labelling performed on the sections using 10 nm gold conjugate as probe. Sections were examined in a JEOL 1400 flash TEM equipped with ATM Nanosprint 12 digital camera and image analysis software.

### Statistics

A Student’s one-tailed *t*-test was used for statistics, and *p* ≤ 0.05 was considered statistically significant. Alongside all data points, mean ± standard deviation is plotted in the figures. All graphs were prepared using GraphPad Prism 9 software.

## Results

### Medium of red-light exposed endothelial cells dilates blood vessel

**Fig. 1:**
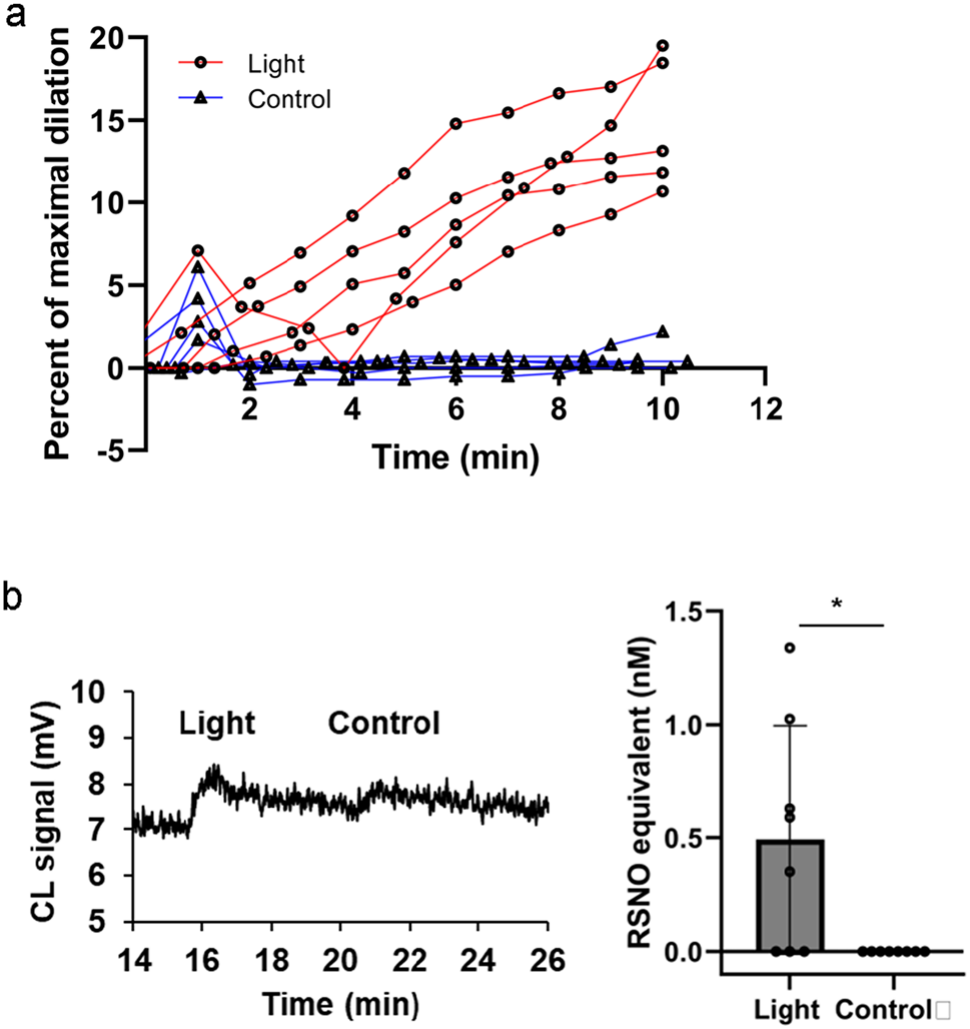
Endothelial cell-derived RSNO-like species dilate ex vivo facial arteries. **a** Facial arteries of C57Bl/6 mice were immersed in media collected from BAECs post irradiation (670 nm, 6 J/cm^2^) and dilation was measured with pressure myography. Vessel diameter was recorded over time and expressed as percent of maximum dilation. *N* = 5 The difference is significant (*p* < 0.05) from 2 min. **b** Media collected from a separate experiment was analyzed for RSNO equivalent species with triiodide-dependent ozone chemiluminescence. Representative trace and quantification of chemiluminescence signal of medium collected from cells exposed to light and dark control. *N* = 8, *p < 0.05

We have previously established that 670 nm light stimulated secretion of a stable vasoactive substance from the endothelium of ex vivo murine facial arteries, which can trigger dilation of a naïve non-irradiated vessel. This substance was detected both in the vessel tissue and in the bath fluid [9] and exhibited the characteristics of RSNO [9, 21]. Moreover, denudation of the vessel abolished vasodilation. To verify the endothelial source of the vasodilatory substance, we replaced the medium of cultured BAECs with vessel bath solution, exposed the cells to 670 nm light, and then treated pressurized non-irradiated vessels with the bath solution removed from the light treated cells. After 10 min, 14.7 ± 0.9% of maximal relaxation was reached (Fig. 1a), which was a significant increase compared to the effect of medium from unexposed cells (0.6 ± 0.8%). In a separate identical experiment, the vessel bath was collected from the cells after light exposure and analyzed. While RSNO levels in the unexposed control samples have not achieved the detection limit, a quantifiable concentration of 0.49 ± 0.50 nM was measured after light exposure (Fig. 1b). These results support the previous findings that the vasodilatory substances are endothelium derived and can be carried over to dilate naïve blood vessels although their levels are minimal.

**Fig. 2:**
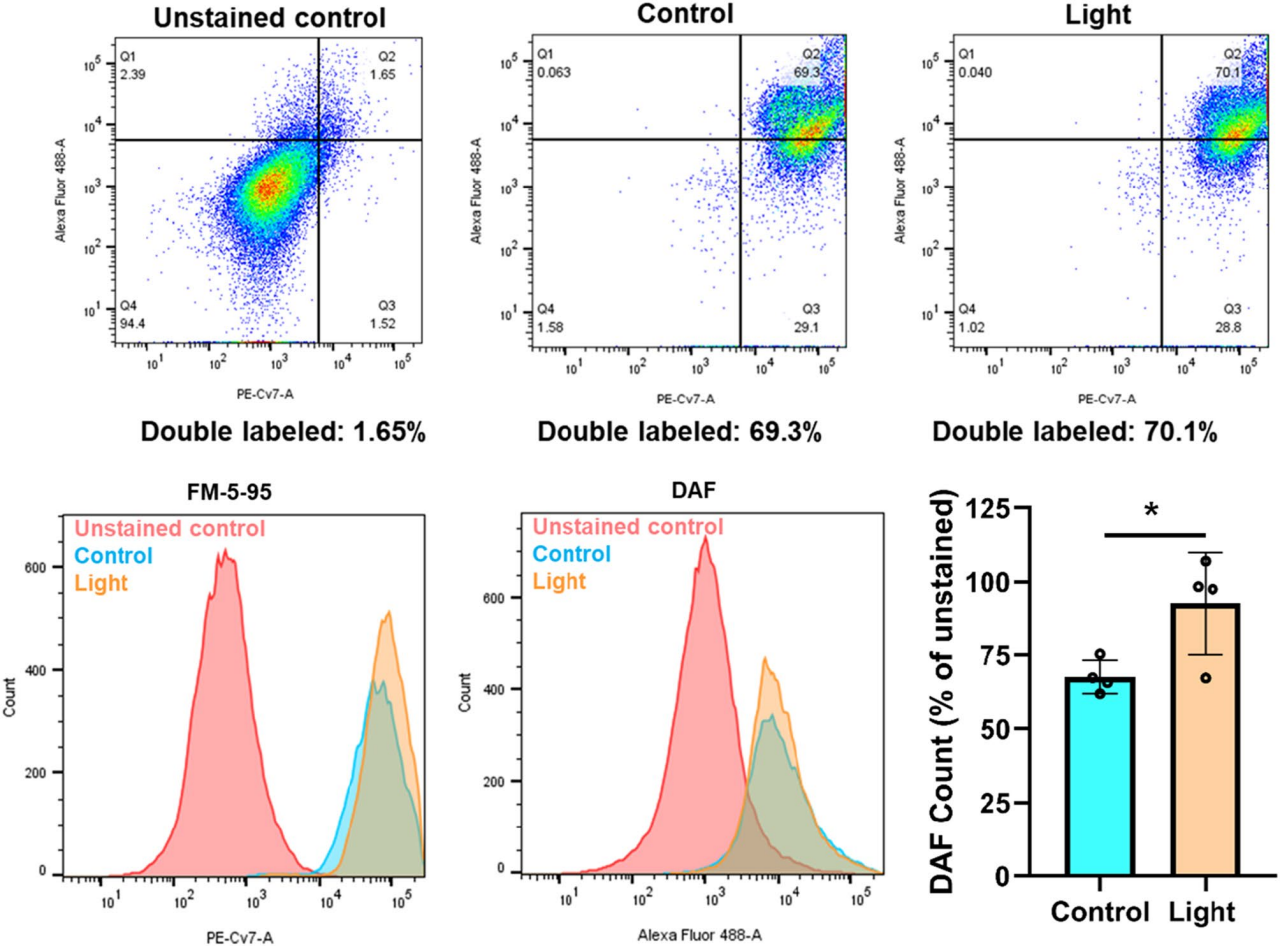
Exosomes contain nitric oxide. BAECs were exposed to 670 nm light (6 J/cm^2^). Flow cytometry was performed on separated extracellular vesicles using DAF for NO labeling, and FM-5–95 as membrane marker. Representative images and quantification. *N* = 4, *p < 0.05

Previous experiments using fluorescence microscopy of the bath particulate from light treated vessels suggested these vasodilatory substances are enclosed in vesicles [10]. Furthermore, red-light facilitates cleavage of RSNO to relaxing factor NO [8], we sought to examine NO levels in the vasodilatory vesicles. We irradiated the cells in the presence of a cell permeable NO indicator DAF-FM diacetate, then separated the vesicles, labeled them with FM 5–95 membrane marker, and analyzed with flow cytometry using AF488 and PE-Cy7 lasers for the DAF and FM 5–95, respectively (Fig. 2). While double labeling in the case of unstained control was minimal (Q2), it showed a 70% overlap in the labeled samples. The light exposure resulted in a significantly higher particle count of NO containing vesicles *vs* dark control.

### Red light stimulates RSNO accumulation in the multi-vesicular bodies

**Fig. 3:**
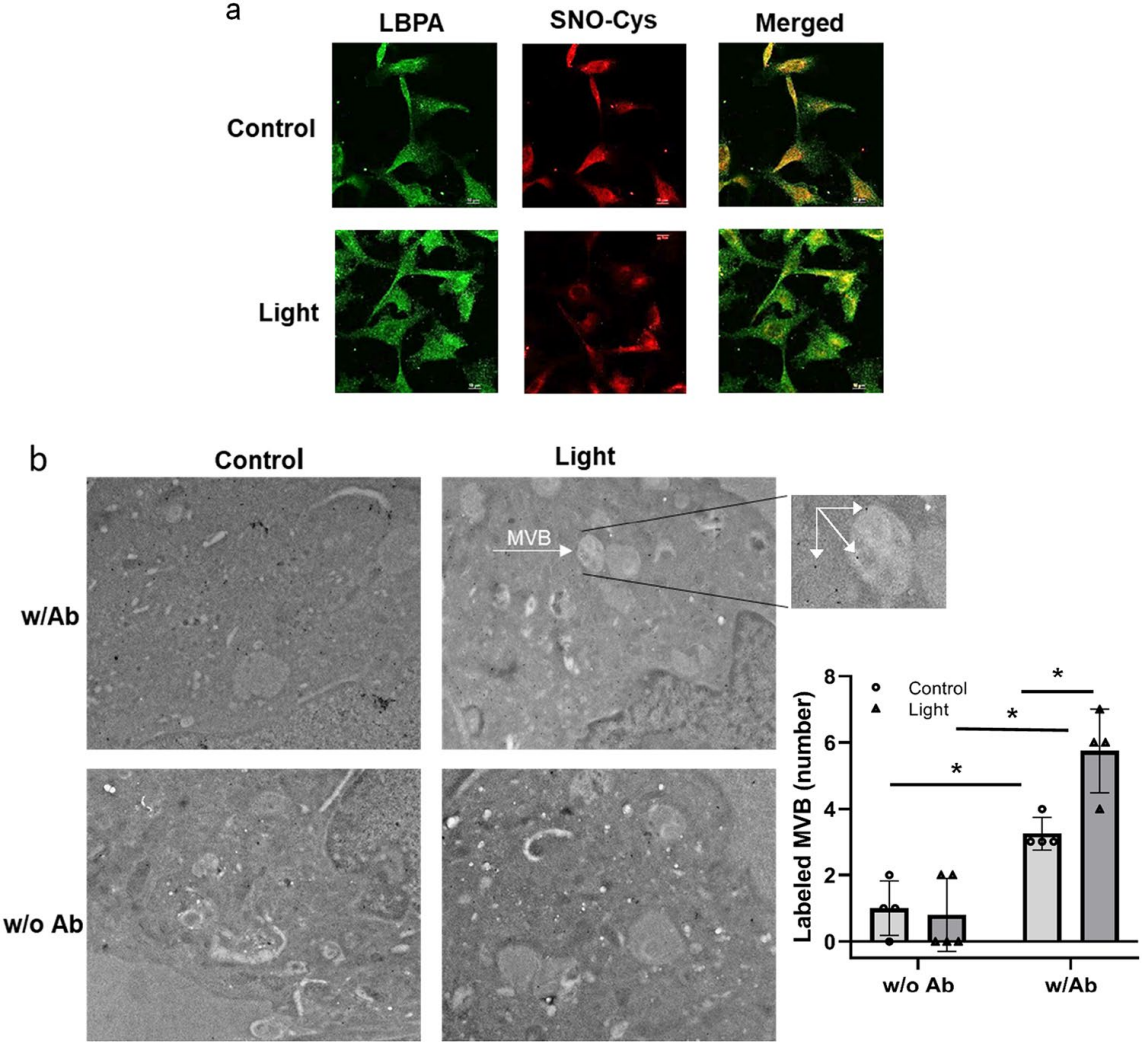
Accumulation of RSNO in Multi Vesicular Body. BAECs were exposed to 670 nm light (6 J/cm^2^). The presence of RSNO in MVBs was assessed **a** immunofluorescence using anti Cys-SNO antibody (red) and MVB marker LBPA (green). **b** Electron microscopy with immunogold labeling, using Cys-SNO antibody (Ab). The gold particles appear as black dots (see inset). *N* = 4

Next, we followed the exocytosis of the major vasodilatory species RSNO. The cellular cargo destined for exocytosis is sorted in the MVB. This organelle is rich in Intra Luminal Vesicles (ILV) which become exosomes upon secretion [12]. Therefore, we investigated whether RSNO accumulates in MVB. Immunocytochemistry displayed a co-localization (yellow) between anti LBPA, a late endosome-specific phospholipid marker [22] (green), and anti Cys-SNO antibodies, which recognizes nitrosated cysteine residues of protein, (green and red, respectively) after light exposure (Fig. 3a). In the control experiments, the RSNO and MBV showed less co-localization i.e. more distinct red and green colors rather than yellow within the cells in the merged panels. The used 100 × magnification enabled to register the color changes. Quantification with electron microscopy by using anti Cys-SNO antibody coupled with immunogold labeling also revealed more RSNO in the MVBs after irradiation (a significant 1.8-fold increase of gold labeling was measured (5.75 ± 1.25 after light exposure vs. 0.8 ± 1.1 in dark control), while in the absence of Cys-SNO antibody, there was no difference in the labeling (Fig. 3b).

### Red light enhances exocytosis

**Fig. 4:**
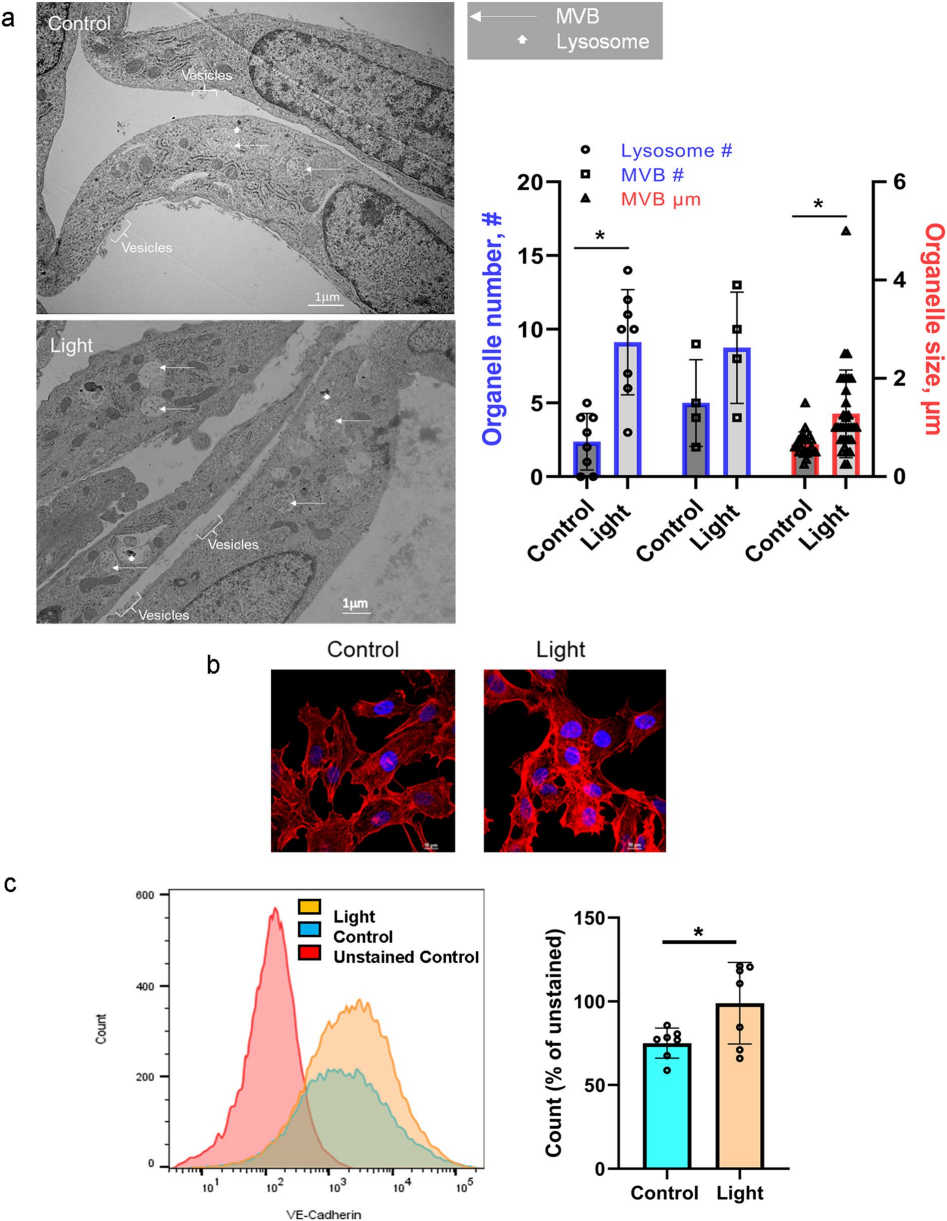
Red light enhances exocytosis. BAECs were exposed to 670 nm light (6 J/cm^2^). **a** Electron microscope imaging. Arrows mark MVB and Lysosomes. Vesicles in the extracellular space are also indicated. *N* = 10. **b** Immunofluorescence staining of F-actin as measured with immunofluorescence (red). The nucleus was stained with DAPI (blue). **c** Flow cytometry was performed on separated extracellular vesicles to detect VE-cadherin. *N* = 7, *p < 0.05

When examining the MVBs with electron microscopy, we found more MVBs in the light exposed cells compared to dark control, however this increase did not reach significance. The size of the MVBs became significantly larger after irradiation (Fig. 4a). Interestingly, 670 nm light resulted in a more abundant number of lysosomes. At the same time, the F-actin distribution showed an increase in stress fibers compared to control. An interruption and disassembly of actin filaments (punctate labeling) is also visible (Fig. 4b). Moreover, we examined the endothelial origin of the exosomes isolated from the medium over the cultured cells. Significantly more particles containing endothelium specific VE-cadherin were counted after red light exposure compared to dark control by using flow cytometry (Fig. 4c).

## Discussion

We have previously recognized that 670 nm energy stimulates dilation of murine facial arteries by stable and transferable RSNO containing microvesicles derived from the endothelium [9, 10]. Here, we confirm red light induces exocytosis of vesicles from cultured endothelial cells which mediate dilation of dissected blood vessels. Our results demonstrate that i) medium of irradiated BAECs contain RSNO that may cause the dilation of the ex vivo facial arteries (Fig. 1), ii) abundance of critical vasodilator RSNO can be assessed in the MVB immediately post irradiation, and red-light-dependent exocytosis can also be detected (Figs. 3, 4), iii) the exosomes contain NO as well (Fig. 2). Interestingly, the light dependent increase of the number of lysosomes raises the possibility of lysosomal exocytosis [23] as well (Fig. 4a).

Exosomes are endocytic vesicles surrounded by lipid bilayer and comprise various cytosolic, and membrane bound proteins, nucleic acids, and cellular metabolites characteristic to the donor cell [12]. Their biogenesis happens in the endosomal system by a complex mechanism and during maturation the load gets enclosed in ILVs and reaches the MVBs that sort the diverse cargos, control the composition, and regulate their secretion by fusing the plasma membrane [12, 13, 24]. The fusion pore expansion is facilitated by a dynamic disassembly and reassembly of F-actin [15, 25] which we found more active by red light exposure. MVBs can also fuse with mature lysosomes to transfer material, but this event commonly leads to the degradation of the transported content [22, 23]. Therefore, the assessed potential lysosomal pathway is most probably a side effect of the light exposure and unrelated to the traffic of vasodilatory vesicles. Right now, we have ongoing studies investigating the molecular mechanisms of red-light facilitated exocytosis.

We found the critical active components of the exosomes are RSNOs, which may derive from cytoplasmic vesicles within vascular endothelial cells [26]. According to latest research [27], RSNOs are also the major constituents of preformed NO stores in vascular smooth muscle which can be activated by various NO derivatives such as nitrite, RSNO, dinitrosyl iron complexes arriving from the endothelium or through red blood cells; and amplify the vasodilatory propensity of the participating external NO species. We have previously implied the presence of NO besides the main product RSNO in the endothelial vesicles released by the 670 nm light [9, 10] probably as a decomposition product of cellular RSNO and other NO precursors [8]. It may be found in the hydrophobic membrane, but with limited concentration because of a potential reaction with also hydrophobic oxygen yields RSNO formation on the thiol residue of some membrane proteins [28, 29], although according to previous findings lipophilic medium does not necessarily enhance *S*-nitrosation [30]. Here, we observe NO together with RSNO leaves the red-light treated endothelial cells making the exosome a critical source of these two species and mediating in the complex mechanism of vasorelaxation. We also suggest that the exiting vesicles may present themselves as intermediaries between the vascular smooth muscle’s internal NO stores [27] and the external vasodilatory molecules. Interestingly, active eNOS was reported in syncytiotrophoblast extracellular vesicles in circulating plasma during pregnancy [31]. This exciting finding may open a new research avenue targeting MVB of endothelial cells. If eNOS is present in MVB, it may contribute to the NO supply leaving the endothelium.

Our previous investigations of longer wavelengths in the red region concluded 740 nm and 830 nm are much less efficient triggers of vasodilation than 670 nm [8]. However, a recent publication shows the infrared (1,460 nm) light is a capable dilator of rat blood vessels ex vivo, via similar NO-dependent mechanisms [32]. The potential applicability of blue light is hindered by its minimal depth of tissue penetration [33].

One limitation of this study is the possible non-specificity of DAF fluorescence which is an indirect measure of NO. The applied Cys-SNO antibody is also prone to some unspecific binding, however, the application of direct detectors, such as NO electrode and a capacitive sensor for RSNO detection [34] cannot be applied in flow cytometry or would lose the advantage of immunofluorescence and electron microscopy which enables to detect the location of critical species. Using two independent techniques for RSNO detection we intended to maximize the reliability of the results.

For therapeutic aspect, there is a possibility that under certain diseased conditions (e. g. diabetes mellitus) the innate RSNO stores are diminished with a potential consequence on the efficacy of light therapy. However, nitrate-rich diet by providing supplemental NO, may help to elevate the levels of stored RSNO. Moreover, we are currently investigating the red-light-dependent role of alternative vasodilatory molecules [35] such as hydrogen peroxide.

## Conclusion

The mechanistic investigations of the red-light induced vasodilation revealed the critical step is the activation of replenishable stores that contain NO precursor species, such as RSNO [20, 36, 37]. Our current results converge on the recognition that 670 nm energy controls RSNO accumulation in the MVB, the destination of vasodilatory cargo before exocytosis, and the exiting vesicles also contain NO. The endothelium derived exosomes may activate the NO stores in the smooth muscle cells and trigger relaxation. The proposed mechanism helps to understand why red-light therapy can serve as a beneficial treatment under certain pathological cardiovascular conditions.

## Data Availability

We submitted during review the statement the data will be made available upon reasonable request.

## Abbreviations

RSNO: *S*-Nitrosothiols
Cys-SNO: *S*-Nitrosocysteine
DAF-FM: 4-Amino-5-methylamino-2′,7′-difluorofluorescein diacetate
DPBS: Dulbecco’s phosphate buffer saline
PSS: Physiological saline solution
HBSS: Hanks’ balanced salt solution
CL: Chemiluminescence
MVB: Multi vesicular body
ILV: Intra luminal vesicle
BAECs: Bovine aortic endothelial cells
LBPA: Lysobisphosphatidic acid
NEM: *N*-Ethylmaleimide
DTPA: Diethylenetriaminepentaacetic acid
SD: Standard deviation
